# Astragaloside IV protects against diabetic nephropathy via activating eNOS in streptozotocin diabetes-induced rats

**DOI:** 10.1186/s12906-019-2728-9

**Published:** 2019-12-05

**Authors:** Yuyan Fan, Hongyu Fan, Bin Zhu, Yilun Zhou, Qingshan Liu, Ping Li

**Affiliations:** 1 Department of Traditional Chinese Medicine, Beijing Tiantan Hospital, Capital Medical University, 119 West Nansihuan Road, Beijing, 100070 People’s Republic of China; 2 Remote Consultation Center, Liaoyang Central Hospital, Liaoyang, Liaoning, 111000 People’s Republic of China; 3 Department of Pharmacy, Beijing Tiantan Hospital, Capital Medical University, Beijing, 100070 People’s Republic of China; 4 Department of Nephrology, Beijing Tiantan Hospital, Capital Medical University, Beijing, 100070 People’s Republic of China; 5 Institute of Chinese Minority Traditional Medicine, Minzu University of China, Beijing, 100081 People’s Republic of China; 6 Department of Pharmacy and Pharmacology, Institute of Clinical Medicine, China-Japan Friendship Hospital, Beijing, 100029 People’s Republic of China

**Keywords:** Astragaloside IV, Diabetic nephropathy, High glucose, Endothelial nitric oxide synthase, Nitric oxide production

## Abstract

**Background:**

Astragaloside IV (AS-IV) was reported to play a role in improving diabetic nephropathy (DN), however, the underlying mechanisms still remain unclear. The aim of the present study is to investigate whether AS-IV ameliorates DN via the regulation of endothelial nitric oxide synthase (eNOS).

**Methods:**

DN model was induced in Sprague-Dawley (SD) male rats by intraperitoneal injection of 65 mg/kg streptozotocin (STZ). Rats in the AS-IV treatment group were orally gavaged with 5 mg/kg/day or 10 mg/kg/day AS-IV for eight consecutive weeks. Body weight, blood glucose, blood urea nitrogen (BUN), Serum creatinine (Scr), proteinuria and Glycosylated hemoglobin (HbA1c) levels were measured. Hematoxylin-Eosin (HE) and Periodic Acid-Schiff (PAS) staining were used to detect the renal pathology. The apoptosis status of glomerular cells was measured by TUNEL assay. The phosphorylation and acetylation of eNOS were detected by western blot. The effects of AS-IV on high-glucose (HG)-induced apoptosis and eNOS activity were also investigated in human renal glomerular endothelial cells (HRGECs) in vitro.

**Results:**

Treatment with AS-IV apparently reduced DN symptoms in diabetic rats, as evidenced by reduced BUN, Scr, proteinuria, HbA1c levels and expanding mesangial matrix. AS-IV treatment also promoted the synthesis of nitric oxide (NO) in serum and renal tissues and ameliorated the phosphorylation of eNOS at Ser 1177 with decreased eNOS acetylation. Moreover, HG-induced dysfunction of HRGECs including increased cell permeability and apoptosis, impaired eNOS phosphorylation at Ser 1177, and decreased NO production, were all reversed by AS-IV treatment.

**Conclusions:**

These novel findings suggest that AS-IV ameliorates functional abnormalities of DN through inhibiting acetylation of eNOS and activating its phosphorylation at Ser 1177. AS-IV could be served as a potential therapeutic drug for DN.

## Background

Diabetic nephropathy (DN) is a common cause of renal failure in many countries, and patients with diabetes mellitus account for about 20–40% of individuals who require renal replacement therapy [1]. A variety of animal models have been established and utilized to investigate DN [2]. Most animal models displayed nephropathic changes containing mild albuminuria, mesangial expansion, and thickening of glomerular basement membrane [3].

It was shown that NO produced by endothelial cells plays a vital role in DN, which led to endothelial dysfunction in diabetes mellitus by inhibiting eNOS and reducing the production and bioavailability of NO [4–6]. A clinical study has reported that low eNOS expression is closely related to the occurrence of DN [7] .Similarly, insufficient eNOS was found to accelerate the occurrence of nephropathy in both type 1 and type 2 diabetic mouse models [8–10]. However, the effective rescue approaches to DN remain unknown.

Astragaloside IV (AS-IV) (C_41_H_68_O_14_, molecular weight = 784.97), a bioactive saponin extracted from the *Astragalus* root, possesses a broad range of pharmacological effects [11–15]. Studies have suggested that AS-IV can alleviate DN by regulating endoplasmic [16], improving mitochondrial damage [11], inhibiting the inflammatory response [17], and relieving oxidative stress [18]. Though AS-IV has been reported to improve endothelial cell dysfunction [19] and alleviate ischemia-reperfusion-induced myocardial injury [20, 21] via up-regulating the eNOS and NO levels, AS-IV can improve DN by activating eNOS is still unknown.

Metabolic memory is one way to explain the difference in the incidence and severity of DN [22]. A previous study has demonstrated that urine protein is present in diabetic rats, whereas the urine protein still appears after rats subcutaneously injected with 5 U/d insulin for 4 weeks with recovering normal blood glucose level in rats [23]. This phenomenon indicates that the urine protein appearance is related to epigenetics in early DN progression and has the characteristics of “metabolic memory”. Ding M et.al have demonstrated that in diabetic cardiomyopathy, increasing silent information regulator 1 (SIRT1) can reduce eNOS acetylation and enhance eNOS phosphorylation and activity [21]. We, therefore, hypothesized that the acetylation of eNOS is related to the “metabolic memory”.

Human renal glomerular endothelial cells (HRGECs) are special capillary endothelial cells, and the high concentration of glucose in the blood will directly lead to the dysfunction and apoptosis of HRGECs, which are the initial factors of DN. In general, the aim of the present study is to investigate whether AS-IV ameliorates DN via the regulation of eNOS in vivo using DN-induced rats model, while the renal protection activities of AS-IV in high glucose (HG)-induced HRGECs were further investigated in vitro.

## Methods

### Animals and drug treatment

Male SD rats, weight of 180-200 g, were obtained from Liaoning Changsheng Biotechnology Company. The study was based on the Guide for the Care and Use of Laboratory Animals and approved by Beijing Tiantan Hospital of Capital Medical University (2017114). The animals were placed in 22 ± 1 °C room, 12 h light/dark cycle, receiving standard chow and water for a week. In this study, the rat received an intraperitoneal injection of streptozotocin (STZ) was used to establish a type I diabetes. Then the rats were received an intraperitoneal injection of either 65 mg/kg STZ (S110910, Aladdin, China) or 0.1 M citrate buffer. Two days after intraperitoneal injection of STZ, rats with a blood glucose level more than 300 mg/dl were considered as diabetic rats and successful establishment of DN model.

**Fig. 1:**
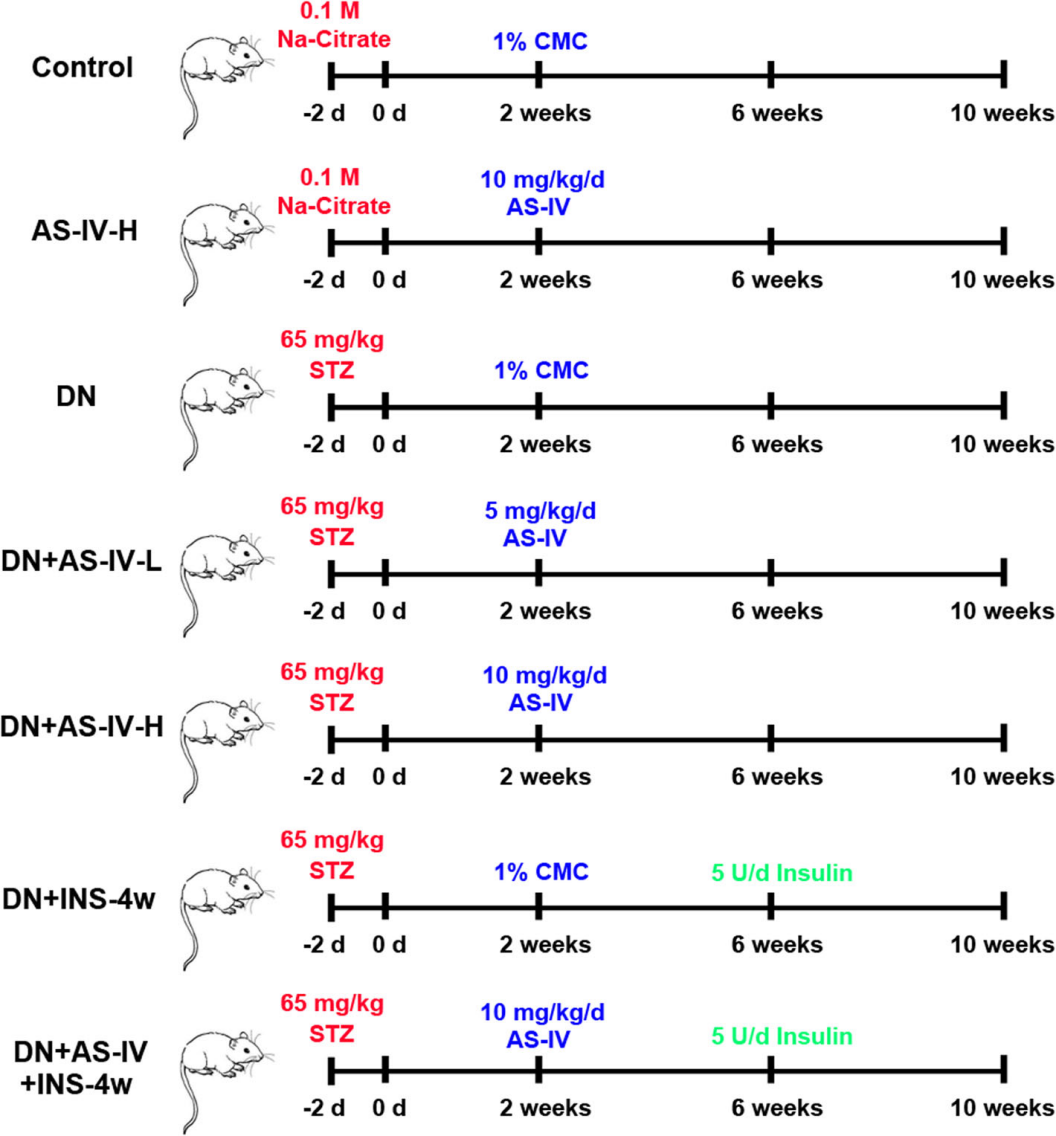
Experimental groups and treatment schedules. AS-IV: Astragaloside IV; STZ: streptozotocin; CMC: carboxymethyl cellulose; Na-Citrate: sodium citrate

Astragaloside IV (AS-IV) (MB1955, Dalian Meilun Biotechnology Co., LTD, Dalian, China) was suspended in 1% carboxymethyl cellulose (CMC) (C104987, Aladdin, China) solution and given to rats by oral gavage. After 2 weeks, 12 healthy rats with the injection of 0.1 M citrate buffer and 30 successful establishment of DN rats were divided into seven groups, each comprising 6 rats, as following (Fig. 1):

Group I (Control): 6 normal rats with the injection of 0.01 M citric acid/0.9% saline solution were treated with 1% CMC for 8 weeks;

Group II (AS-IV-H): 6 normal rats with the injection of 0.01 M citric acid/0.9% saline solution were treated with 10 mg kg^− 1^ d^− 1^ for 8 weeks;

Group III (DN): 6 DN rats were treated with 1% CMC for 8 weeks;

Group IV (DN + AS-IV-L): 6 DN rats were treated with AS-IV at 5 mg kg^− 1^ d^− 1^ for 8 weeks;

Group V (DN + AS-IV-H): 6 DN rats were treated with AS-IV at 10 mg kg^− 1^ d^− 1^ for 8 weeks;

Group VI (DN + INS-4w): 6 DN rats were treated with 1% CMC for 8 weeks, and at the beginning of the seventh week, the rats were subcutaneous injected with 5 U/d Insulin (Baiying Biotechnology Company) for 4 weeks;

Group VII (DN + AS-IV + INS-4w): 6 DN rats were treated with AS-IV at 10 mg kg^− 1^ d^− 1^ for 8 weeks, and at the beginning of the seventh week, the rats were subcutaneous injected with 5 U/d Insulin for 4 weeks;

After 8 weeks treatment, the rats were sacrificed by intraperitoneal injection of pentobarbital sodium at 200 mg/kg and the kidneys were harvested for further study. The blood glucose of each group was measured from the third week to the tenth week. The body weight of each group was measured at the end of tenth week.

### Metabolic analysis

Fasting blood glucose levels were monitored with Glucometerusing one drop of tail blood every week after injection with STZ 2 weeks. Blood urea nitrogen (BUN) was measured using the Urea Assay Kit (C013, Nanjing Jiancheng Bioengineering Institute, Nanjing, China). HbA1c was measured using Glycosylated hemoglobin assay kit (A056, Nanjing Jiancheng Bioengineering Institute, Nanjing, China). Proteinuria was measured using a Urine protein test kit (C035, Nanjing Jiancheng Bioengineering Institute, Nanjing, China). Serum creatinine (Scr) was measured using a Creatinine (Cr) Assay kit (C011, Nanjing Jiancheng Bioengineering Institute, Nanjing, China). The NO levels from serum and renal tissue were detected to evaluate the oxidative stress and the magnitude of vascular damage in diabetic rats. This NO was quantified by Nitric Oxide (NO) assay kit (A013, Nanjing Jiancheng Bioengineering Institute, Nanjing, China) and BCA Protein Quantification Kit (PC0020, Solarbio, Beijing, China).

### Kidney histopathological examination

To determine the histopathological changes in kidney, the kidneys from rats were staining by ematoxylin-Eosin (HE). The kidney of the rat was fixed in 10% formalin and embedded in paraffin. After deparaffinization and rehydration, 5 µm thickness kidney sections were measured by Hematoxylin-Eosin Staining (WLA051a, Wanleibio, China) as described by the manufacturer. Finally, the sections were observed and photographed by an optical microscope (DP73, OLUMPUS, Japan).

The kidney tissues were dehydrated, embedded in paraffin, sectioned at 5 μm thickness, and stained by Periodic Acid-Schiff (PAS) (BA-4114, Baso, China) stain. Each sample slice was observed under the microscope (DP73, OLUMPUS, Japan) at a magnification of 200 × .

### Cell culture

Human renal glomerular endothelial cells (HRGECs) were purchased from Procell and maintained in Human glomerular endothelial cell culture medium in a humidified incubator containing 5% CO_2_ at 37 °C. L-NAME was a kind of NO synthesis (NOS) inhibitor. HRGECs were treated with either (a) without treatment (served as controls) (b) 30 mM Mannitol (c) 30 mM glucose (d) pre-stimulation with 50 μM AS-IV for 12 h, followed by adding with 30 mM glucose, (e) pre-stimulation with 100 μM L-NAME (HY-18729A, CEM, USA) for 12 h, 50 μM AS-IV for 12 h, and followed by adding with 30 mM glucose.

### MTT assay

To rule out the possibility of cell injury by high glucose exposure, the cell ability of HRGECs was determined by MTT assay. Briefly, the cells at a density of 8 × 10^3^ / well were cultured for 48 h in media containing among normal glucose (Control), 30 mM mannitol (Mannitol) or 30 mM glucose (High glucose; HG). The HRGECs were treated with 50 μM AS-IV for 12 h and then cultured in media containing 30 mM glucose for 48 h (HG + AS-IV). The HRGECs were treated with 100 μM L-NAME and 50 μM AS-IV for 12 h and then cultured in media containing 30 mM glucose for 48 h (HG + AS-IV + L-NAME). The final volume of cell culture medium in each well was 200 μl. Four hours after the end of culture, human glomerular endothelial cell culture medium (Procell, CM-H061) containing 0.5 mg/ml MTT was added to each well, at 4 h, culture media were removed and 150 μl DMSO (Beyotime, ST038) solution was added. Finally, the optical density was measured at 570 nm with a microplate reader (BIOTEK, ELX-800).

### Endothelial cell permeability

Permeability was examined by clearance of albumin across endothelial monolayers. Monolayer cells were seeded on the inserts of Transwell (3422, Corning, USA). After washing, the monolayer cells were incubated with PBS containing 0.035% trypan blue and 0.8% BSA and incubated at 37 °C for 1 h. Absorbance at 590 nm was measured from the lower well fluids.

### TUNEL assay

The HRGECs were fixed with 4% paraformaldehyde and permeabilized with 0.1% Triton X (ST795, Beyotime, China). The DNA fragmentation was determined by Terminal deoxynucleotidyl transferase (TdT) -mediated dUTP nick end labeling (TUNEL) via In Situ Cell Death Detection Kit (11,684,955,910, Roche, Swiss) as described by the manufacturer. DAPI is used to locate the nuclei of the cells. Fluorescent images were obtained using the microscope (DP73, OLUMPUS, Japan).

TUNEL staining was performed using a peroxidase In Situ Cell Death Detection Kit (11,684,955,910, Roche, Swiss). Tissue sections were treated with 3% hydrogen peroxide and added the equilibration buffer. Then the sections were treated with TUNEL reaction buffer for 60 min at 37 °C. Specimens were then treated with Converter-POD for 30 min at room temperature and developed with 3,30-diaminobenzidine (DAB) substrate. Sections were counterstained with hematoxylin (H8070, Solarbio, China), rinsed, dehydrated, and mounted. Each slice was observed under a microscope (DP73, OLUMPUS, Japan); the DAB staining intensity was taken to indicate the extent of apoptosis detected by TUNEL.

### Western blot

Samples (20 μg) were subjected to SDS–PAGE analysis and electrotransferred to membranes (PVDF, IPVH00010, Millipore, USA), washed with TBST, and then incubated with Rabbit Anti-eNOS antibody (1:1000; Bs-20608R, Bioss, China), eNOS (phospho Ser1177) antibody (1:1000; GTX129058, Gene Tex, USA), eNOS (phospho Thr495) antibody (1:1000; PA5–17706, ThermoFisher, USA) or GAPDH Antibody (1:10000; 60,004–1-Ig, Proteintech, China) at 4 °C overnight. After washing with TBST, membranes were incubated with Goat Anti-rabbit IgG/HRP antibody (1:3000; SE134, Solarbio, USA) or Goat Anti-Mouse IgG/HRP antibody (1:3000; SE131, Solarbio, USA). Finally, the results were visualized by an enhanced fluoro-chemiluminescent system (ECL, PE0010, Solarbio, China).

### eNOS acetylation expression

The expression of eNOS acetylation was determined by co-immunoprecipitation assay. eNOS (crosslinked to magnetic beads (P2012, Beyotime, China) for extraction) was immunoprecipitated from renal tissue lysate, and the primary antibody for acetyl-lysine (1:200, SC-81623, Stana, USA) was employed to detect the association of the acetyl-lysine with eNOS by using immunoblotting.

### Statistical analysis

Data are presented as mean ± standard deviation (SD) and were analyzed using GraphPad Prism 7.0 (GraphPad Software Inc., USA). The Student’s t-test or one-way analysis of variance (ANOVA) followed by Bonferroni’s *post-hoc* test was used for the comparison between two groups or among more than two groups. *P* < 0.05 was considered a statistically significant difference.

## Results

### AS-IV improves metabolic parameters of DN rats

**Fig. 2:**
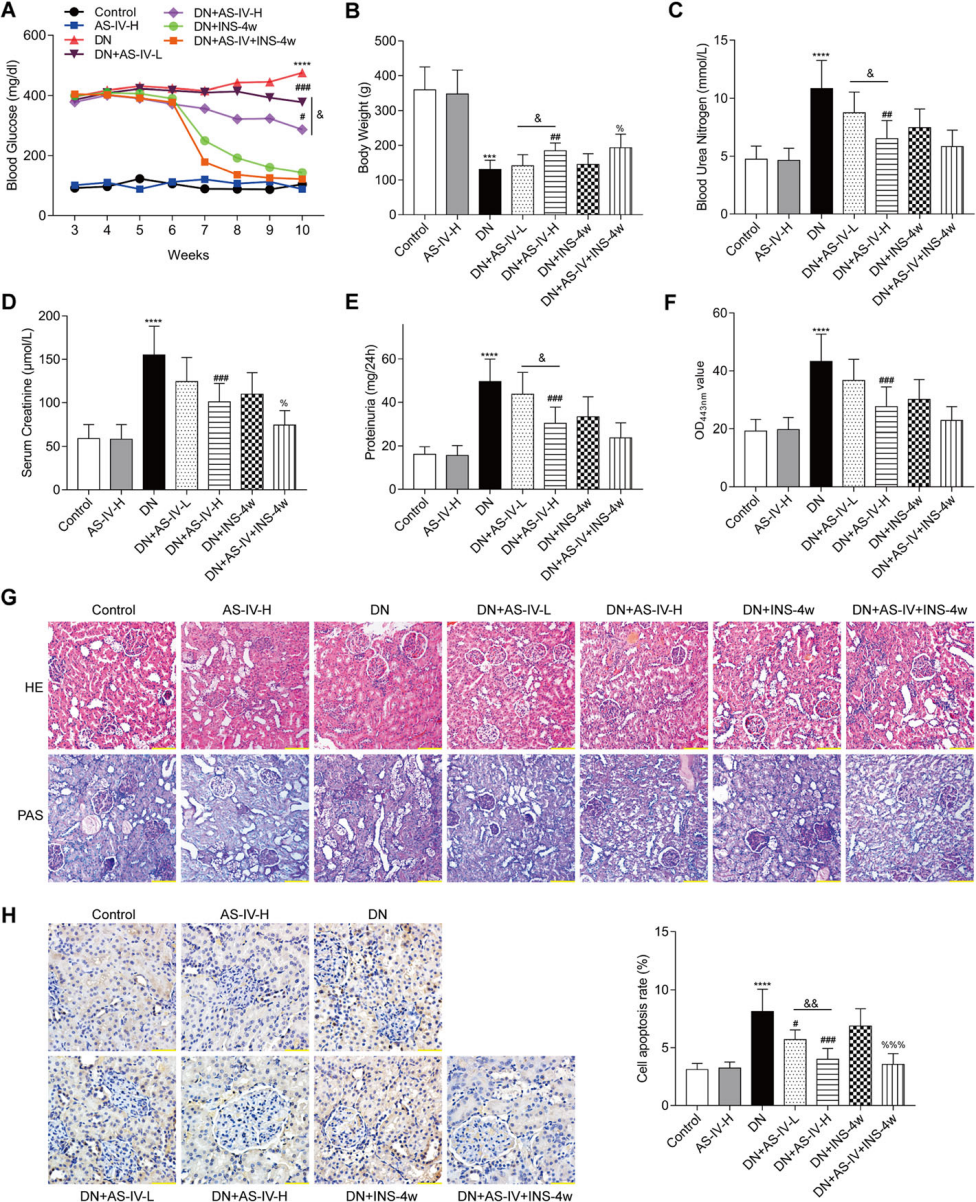
Effects of AS-IV on metabolic parameters in kidney damage rats. **a** Fasting blood glucose was recorded every week after injecting STZ two weeks. **b** Body weight (**b**), Blood urea nitrogen (**c**), Scr serum creatinine (**d**), proteinuria (**e**), HbA1c (**f**) of rats was measured at the end of the study. **g** Kidney sections from each group of rats were detected by HE and PAS staining, bar = 100 μm. H Representative images of TUNEL staining, bar = 50 μm. Data are expressed as mean ± SD (*n* = 6). ****p < 0.0001, ***p < 0.001 vs. control; ###*p* < 0.001 vs. DN, ##*p* < 0.01; %*p* < 0.05 vs DN + ING-4w. &&*p* < 0.01, &*p* < 0.05

The blood glucose of healthy rats received AS-IV treatment could not change. Two weeks after STZ injection, diabetic rats showed a significant increase in fasting blood glucose levels when compared with the control rats. Treatment of diabetic rats with AS-IV or INS-4w significantly reduced fasting blood glucose levels (Fig. 2a). Compared to DN rats, both the AS-IV-H and AS-IV-L treatment could reduce the blood glucose. More importantly, compared to AS-IV-L rats, AS-IV-H rats showed a better beneficial effect on reduction of blood glucose, suggesting that the 10 mg kg^− 1^ d^− 1^ dose of AS-IV might be the better choice for DN treatment. Compared to control rats, diabetic rats showed a significant (*P* < 0.001) decrease in body weight at the end of the study, whereas the AS-IV-H rats remained normal level (Fig. 2b). These results showed that diabetic rats could decrease body weight and increase blood glucose, however, the AS-IV treatment could not influence body weight and blood glucose. High levels of blood urea nitrogen (BUN) increased risk of insulin use, and serum creatinine (Scr) concentration is inversely related to the incident of diabetes [24, 25]. The BUN and Scr analysis showed AS-IV significantly reduced BUN and Scr levels in diabetic rats treated with 5 mg kg^− 1^ d^− 1^ or 10 mg kg^− 1^ d^− 1^ AS-IV or 5 U/d INS-4w as depicted in Fig. 2c and Fig. 2d. These results suggested that AS-IV-H could decrease the risk of insulin use and AS-IV-L could inhibit the incident of diabetes. Figure 2e shows AS-IV significantly decreased proteinuria levels in diabetic rats treated with 5 mg kg^− 1^ d^− 1^ or 10 mg kg^− 1^ d^− 1^ AS-IV or 5 U/d INS-4w. Moreover, the AS-IV could reduce the proteinuria level in diabetic rats compared with single treated with INS-4w rats (Fig. 2e). The anti-hyperglycemic effect of AS-IV was confirmed by the level of HbA1c at OD_443nm._ Compared with the control rats, the diabetic rats exhibited a significant (*P* < 0.001) increase in HbA1c. Diabetic rats received AS-IV or INS-4w showed a significantly attenuated in HbA1c (Fig. 2f). The blood glucose was decreased by AS-IV may be caused by the decreasing of HbA1c. The representative photomicrographs of rat kidneys in PAS-stained and HE-stained from the seven groups were shown in Fig. 2g. Eight weeks after treatment, the diabetic rats demonstrated a mesangial matrix expansion in glomeruli compared with control rats. In contrast, compared with the untreated diabetic rats, AS-IV treated rats showed attenuated mesangial expansion. Notably, the mesangial matrix expansion of glomeruli in AS-IV-H rats could be alleviated compared to AS-IV-L rats. The decreased quantity of cells in the glomerulus is considered as one of the main causes of diabetic nephropathy. On the one hand, the diabetic rats showed a significant increased in cell apoptosis when compared with the control rats (*P* < 0.0001). On the other hand, AS-IV treatment also reversed the kidney cell apoptosis in diabetic rats (Fig. 2h). Similarly, compared to AS-IV-L treatment, AS-IV-H treatment significantly decreased the cell apoptosis rate. Thus, AS-IV improves metabolic parameters of DN rats, especially the 10 mg kg^− 1^ d^− 1^ dose of AS-IV.

### AS-IV treatment increased eNOS activity in diabetic rats

**Fig. 3:**
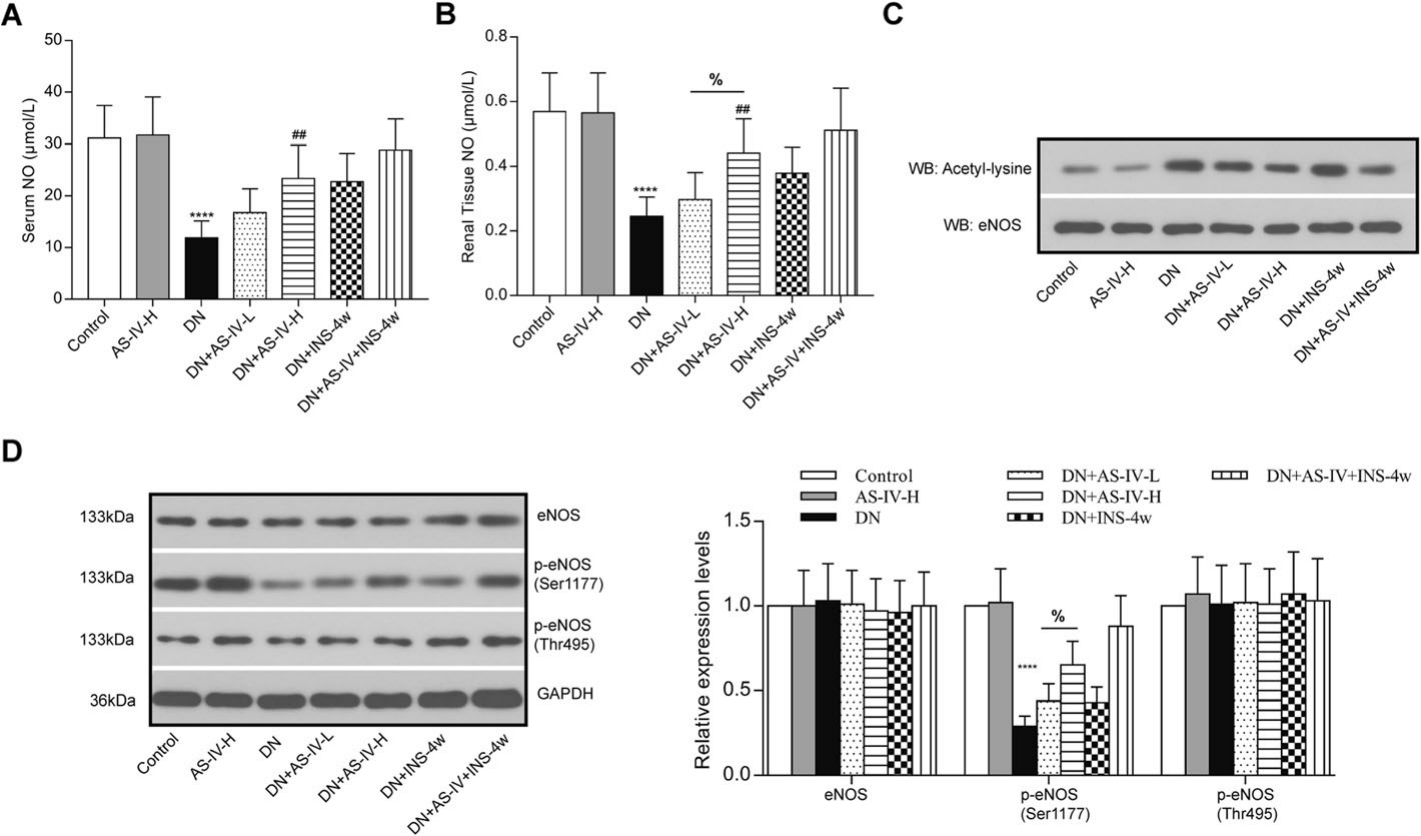
AS-IV reversed high glucose-induced endothelial nitric oxide synthase (eNOS) Serum NO (**a**) and Renal Tissue NO (**b**) after AS-IV treatment in rats were detected by Nitric Oxide (NO) assay kit. *n* = 6 per group. Results are represented as mean and SD. **c** representative blot image of acetylated eNOS. **d** Western blot analysis for the expressions of eNOS, p-eNOS (Ser1177) and p-eNOS (Thr495). ****p < 0.0001 vs. control; ##*p* < 0.01 vs. DN. &*p* < 0.05

STZ treatment reduced serum NO level and renal tissue NO level, both AS-IV-L and AS-IV-H treatment had increased serum and renal tissue NO levels compared to the diabetic rats (Fig. 3a and b). More importantly, AS-IV-H treatment remarkably promoted the renal tissue NO level compared to AS-IV-L treatment. AS-IV significantly increased eNOS activity and attenuated eNOS acetylation in diabetic renal (Fig. 3c). In addition, under the treatment of INS-4w, the AS-IV could provide a better therapeutic effect for DN in diabetic rats. NO production was confirmed to be increased by phosphorylation of eNOS. Intra-renal eNOS, phospho-Thr495 eNOS, and phospho-Ser1177 eNOS levels were determined using Western blot analysis. There were no differences in eNOS and phospho-Thr495 eNOS among the experimental and control groups regardless of adding AS-IV or INS-4w. High glucose decreased eNOS phosphorylation at Ser1177 with no significant change at Thr495. Phospho-Ser1177 levels were decreased in diabetic rats, which were reversed after AS-IV treatment, especially AS-IV-H treatment. Compared with INS-4w + DN rats, the treatment of AS-IV showed aggravated phosphorylation in Phospho-Ser1177 (Fig. 3d). These results suggested that AS-IV enhanced eNOS activity and NO production by inhibiting acetylation and promoting phosphorylation at Ser1177 of eNOS in diabetic rats.

### AS-IV reversed HG-induced cell permeability and apoptosis in HRGECs

**Fig. 4:**
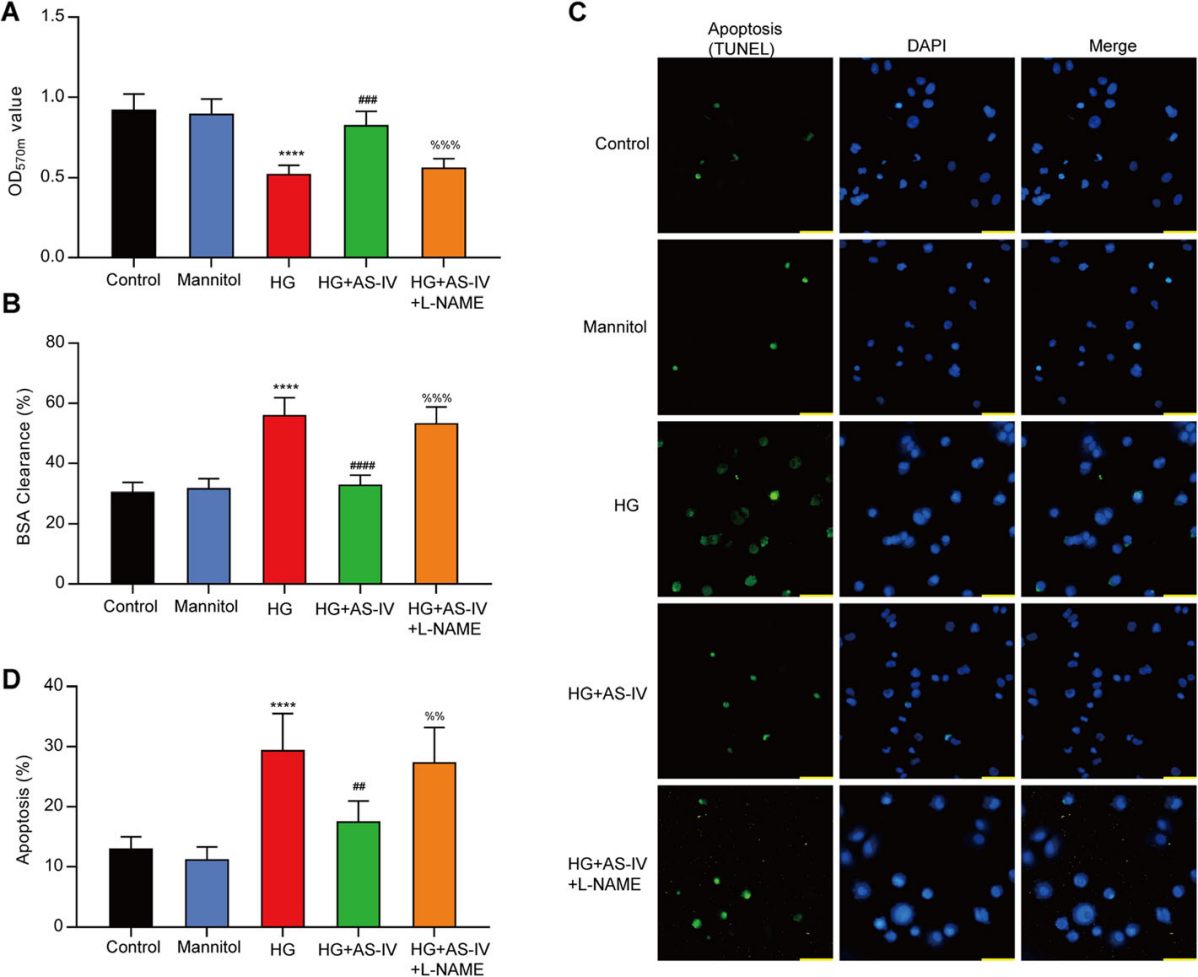
High glucose increased cell permeability and apoptosis in HRGECs, whereas these effects were inhibited by AS-IV. **a** Cell viabilities in HRGECs were matured by MTT assay. All results were expressed as mean ± SD (*n* = 5). **b** The permeability of HRGECs was evaluated by BSA clearance as described in methods. **c** HRGECs were stained using TUNEL or DAPI, bar = 50 μm. **d** Effect of HG on apoptosis in HRGECs was detected by TUNEL assay. ****p < 0.0001 vs. control; ###*p* < 0.001, ##*p* < 0.01 vs. HG; %%%*p* < 0.001, %%*p* < 0.01 vs HG + AS-IV

Previous studies indicated that the incubation of cultured HRGECs with HG for 48 h demonstrated significant alterations. L-NAME was proved to inhibit NO synthesis (NOS). The results of the MTT assay showed incubation in the HG decreased cell proliferation in HRGECs compared to the control cells, which reversed by AS-IV. After adding L-NAME, the capability of cell proliferation was further decreased (Fig. 4a). The cell permeability was detected by the BSA clearance assay, and the results revealed that AS-IV attenuated HG-induced permeability (Fig. 4b). Moreover, the results from the TUNEL assay showed that AS-IV blocked HG-induced apoptosis of HRGECs (Fig. 4c&d). These findings suggested that HG increased BSA permeability and apoptosis in HRGECs, but these effects were restored by AS-IV treatment.

### AS-IV counteracted HG-induced suppression of eNOS in HRGECs

**Fig. 5:**
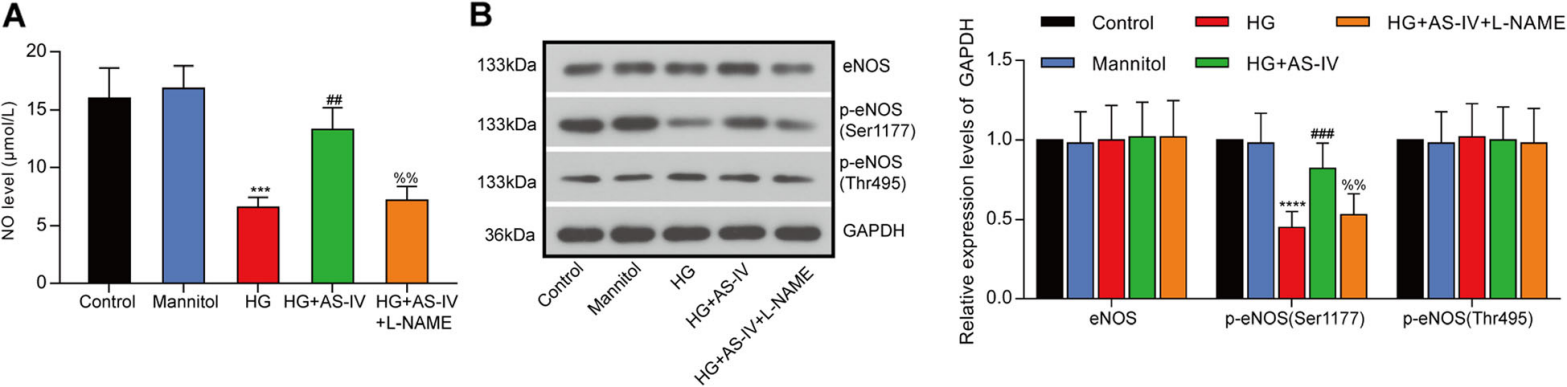
AS-IV reversed HG-induced eNOS impairment in HRGECs. **a** The NO producing ability in HRGECs after high glucose treatment was assessed by Nitric Oxide (NO) assay kit. All results were expressed as mean ± SD (*n* = 3). **b** The expressions of eNOS, p-eNOS (Ser1177) and p-eNOS (Thr495) after high glucose treatment in HRGECs were detected by western blot. ****p < 0.0001, ***p < 0.001 vs. control; ###*p* < 0.001, ##*p* < 0.01 vs. HG; %%*p* < 0.01 vs HG + AS-IV

HG-induced reduction of NO content was restored by AS-IV treatment (Fig. 5a). To reveal the effects of AS-IV on the activation of eNOS, the phosphorylation levels of Thr495 and Ser1177 of eNOS were determined by western blot. HG decreased eNOS phosphorylation level at Ser1177 and these were reversed by AS-IV treatment (Fig. 4b). Thus, AS-IV could activate eNOS and increase NO level.

## Discussion

Our results indicate that treatment with AS-IV apparently protected the kidney from diabetic-induced injury, as evidenced by downregulation of BUN, Scr, proteinuria, HbA1c and expanding mesangial matrix. AS-IV treatment also significantly promoted the synthesis of NO in serum and renal tissues and promoted the phosphorylation of eNOS at Ser 1177 with decreased eNOS acetylation. HRGECs are special capillary endothelial cells, and the high concentration of glucose in the blood will directly lead to the dysfunction and apoptosis of HRGECs, which are the initial factors of DN. HG-induced dysfunction of HRGECs was also alleviated by AS-IV treatment. To the best of our knowledge, this is the first study demonstrating that AS-IV attenuated DN in diabetic rats through alleviating eNOS acetylation and intensifying the phosphorylation of eNOS at Ser 1177.

DN could be caused by multiple factors, such as glomerular cell growth and extracellular matrix accumulation [26]. AS-IV is a small molecular saponin and has diverse pharmacological activities, which has widely used in traditional medicine in China. AS-IV suppressed podocytes apoptosis through miR-378/TRAF5 signaling pathway, thereby inhibiting the development of DN [27]. In diabetic rats, AS-IV ameliorates DN by inhibiting ILK expression and restoring integrin α3β1 expression [28]. Liu X et.al have demonstrated that AS-IV could restore the mitochondrial quality control network of diabetic mice [11]. In addition, AS-IV is effective in reducing blood glucose levels, reducing urine albumin excretion and delaying the development of DN [29]. Studies also show that AS-IV reduces proteinuria and attenuates diabetes, which is related to the reduction of ER stress [30]. In this study, AS-IV reduced blood glucose levels and alleviated the proteinuria level in STZ-induced diabetic rats, which is similar to other researches. More importantly, AS-IV recovery the NO production in STZ-induced diabetes rats via decreasing the acetylation and increasing phosphorylation at Ser 1177 of eNOS. These findings may provide a new therapeutic target against nephropathy diseases.

eNOS plays a role in regulating angiogenesis and repairing endothelial cell damage in renal diseases [31–34]. Providing a precursor for the production of NO significantly improved the function of glomerular endothelial cells and the process of glomerular injury in mice [35]. Moreover, diabetic mice with eNOS deficiency showed significant nephropathy changes with significant progressive DN characteristics, including significant proteinuria, nodular glomerulosclerosis, angiolysis, and hyaline arteriolar degeneration. Also, eNOS deficiency reduced oxidative stress and tubulointerstitial fibrosis in mice [3]. Thus, the cause of DN might be the inactivation of the eNOS-NO axis [36]. In the progression of DN, the release of endothelium-derived NO requires the activation of eNOS. Moreover, the activity of eNOS can be regulated by phosphorylation. The serine/ threonine protein kinase Akt has been shown to increase human eNOS activity by Akt phosphorylation at Ser1179 and then lead to increase NO production, whereas mutant eNOS (Ser 1179A and Ser 1177) is resistant to phosphorylation and Akt-dependent activation [37, 38]. Reduced phosphorylation of eNOS at Ser1179 has previously been reported in diabetic rats [39]. A previous study has demonstrated that in both diabetic glomeruli and high-glucose GEnCs, eNOS phosphorylation was impaired at Ser1179, suggesting that the phosphorylation of eNOS at Ser1179 was related to diabetic nephropathy [35]. Moreover, Akt-dependent phosphorylation of eNOS at Ser1179 has been proved to be essential for endothelium-dependent relaxation [40]. As for the acetylation, a recent report has highlighted the role of eNOS/iNOS and the NO production in the renal diseases including DN, which are controlled by histone acetylation [41]. Moreover, the histone deacetylation inhibition decreased eNOS mRNA while paradoxically increasing the activity of its promoter, suggesting the induction of an eNOS mRNA-destabilizing factor [42]. In the present study, AS-IV treatment also significantly promoted the phosphorylation of eNOS at Ser 1177 but decreased the eNOS acetylation to induce the synthesis of NO. These findings can provide evidence that AS-IV protects renal against DN by the activation of eNOS in vivo.

Notably, AS-IV up-regulates eNOS expression and NO production and triggered the eNOS/NO/ONOO-pathway by inhibiting CAV-1 [19, 43]. It has been shown that AS-IV significantly increased eNOS expression and NO production, whereas L-NAME partially abolished the effect of AS-IV [44]. To further clarify the role of AS-IV in DN through activating eNOS, the eNOS inhibitor, L-NAME, was applied to HG-treated HRGECs. The results showed that co-treatment with L-NAME not only promoted apoptosis but also blocked NO production. These data further confirmed that AS-IV rescues the diabetic rat nephropathy via an eNOS-dependent mechanism.

Additionally, our results demonstrate that when compared to insulin single treatment group, the proteinuria in the AS-IV co-treated group is reduced, however, the differences are not statistical significance. We speculate that this may due to the low AS-IV treatment concentration or insufficient treatment time. Although the current results are not enough to prove that AS-IV can relieve DN by changing the metabolic memory of diabetic, however, considering the inhibitory effect of AS-IV on eNOS acetylation, we still believe that AS-IV can improve metabolic memory. These will be investigated in our future research.

## Conclusion

In conclusion, this study indicates that AS-IV can ameliorate DN through the activating of phosphorylation and inhibiting acetylation of eNOS. This novel finding provides support for the role of AS-IV in the treatment of DN based on targeting the regulation of eNOS activity and NO production.

## Data Availability

Available from the corresponding author on reasonable request.

## Abbreviations

AS-IV: Astragaloside IV
BUN: Blood urea nitrogen
Cr: Creatinine
DN: Diabetic nephropathy
eNOS: Endothelial nitric oxide synthase
HE: Hematoxylin-Eosin
HG: High-glucose
NO: Nitric oxide
PAS: Periodic Acid-Schiff
STZ: Streptozotocin
